# 159. Diagnostic Utility of Nasal Methicillin-resistant *Staphylococcus aureus* Polymerase Chain Reaction Testing in Head and Neck Infection

**DOI:** 10.1093/ofid/ofad500.232

**Published:** 2023-11-27

**Authors:** Jake Smith, Bailee Cummings, Ryan K Dare

**Affiliations:** University of Arkansas for Medical Sciences, Little Rock, Arkansas; Tulane University, Little Rock, Arkansas; University of Arkansas for Medical Sciences, College of Medicine, Little Rock, Arkansas

## Abstract

**Background:**

Methicillin-resistant *Staphylococcus aureus* (MRSA) is a clinically important pathogen that is responsible for significant morbidity and mortality worldwide. MRSA is a colonizer of human nasal passages. Prior studies have shown that lack of detection of MRSA via polymerase chain reaction (PCR) of nasal swab specimens can accurately predict absence of MRSA in certain infections. Currently, guidelines posed by the Infectious Disease Society of America make no mention of the utility of MRSA PCR swabs for empiric antibiotic choice in facial abscess. This study investigated the diagnostic performance characteristics of MRSA nasal PCR in patients undergoing various ear, nose, and throat surgeries.

**Methods:**

A retrospective analysis was performed at UAMS looking at adult patients with; (1) Ear nose and throat (ENT) surgeries, (2) collection of nasal MRSA PCR, (3) operating room (OR) culture data from the surgery; all within the same admission. OR culture data was used as the reference standard for presence of MRSA in surgical isolates. MRSA PCR was the index test of interest. The primary outcome was the diagnostic performance characteristics of the assay. Secondary outcomes included diagnostic performance characteristics of PCR for methicillin-sensitive staphylococcus aureus (MSSA).

**Table 1:**
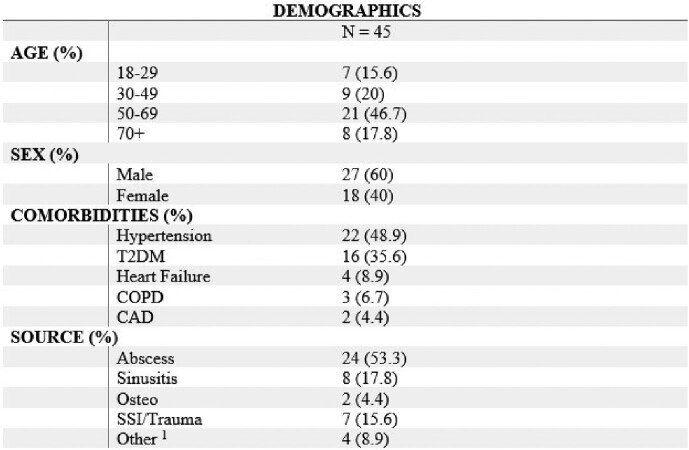
Baseline demographics and clinical characteristics of study cohort

1: Includes necrotizing fasciitis, neck mass, tracheo-esophageal fistula repair, and tracheal stenosis repair. Abbreviations: T2DM - Type 2 diabetes mellitus, COPD - chronic obstructive pulmonary disease, CAD - coronary artery disease, SSI - surgical site infection

**Results:**

In 45 patients meeting inclusion criteria, MRSA was isolated in 4.4% of OR cultures. When compared to OR cultures, MRSA nasal PCR demonstrated 100% sensitivity and 93% specificity with a positive predictive value of 40% and a negative predictive value (NPV) of 100%. MSSA PCR testing showed a lower negative predictive value of 97.1% for presence of MRSA or MSSA.

**Table 2:**
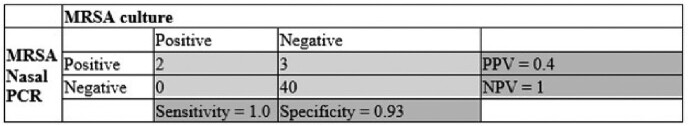
Diagnostic characteristics of MRSA nasal PCR assay

**Table 3:**
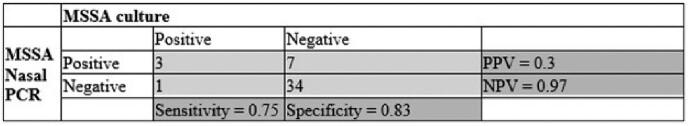
Diagnostic characteristics of MSSA nasal PCR assay

**Conclusion:**

In patients undergoing ENT surgeries, MRSA nares PCR assay has a high NPV for presence of MRSA and can be used to help guide vancomycin de-escalation.

**Disclosures:**

**All Authors**: No reported disclosures

